# Drug repositioning as a promising approach for the eradication of emerging and re-emerging viral agents

**DOI:** 10.1007/s11030-025-11131-8

**Published:** 2025-03-18

**Authors:** Marwa Almulhim, Abdolmajid Ghasemian, Mojtaba Memariani, Farnaz Karami, Asmaa S. A. Yassen, Athanasios Alexiou, Marios Papadakis, Gaber El-Saber Batiha

**Affiliations:** 1 Department of Internal Medicine, College of Medicine, Jouf University, Sakaka, Saudi Arabia; 2 Noncommunicable Diseases Research Center, Fasa University of Medical Sciences, Fasa, Iran; 3 Department of Mycobacteriology and Pulmonary Research, Pasteur Institute of Iran, Tehran, Iran; 4 Microbiology Research Center (MRC), Pasteur Institute of Iran, Tehran, Iran; 5 Pharmaceutical Organic Chemistry Department, Faculty of Pharmacy, Suez Canal University, Ismailia, 41522 Egypt; 6 University Centre for Research & Development, Chandigarh University, Chandigarh-Ludhiana Highway, Mohali, Punjab India; 7 Department of Science and Engineering, Novel Global Community Educational Foundation, Hebersham, NSW 2770 Australia; 8 Department of Surgery II, University Hospital Witten-Herdecke, University of Witten-Herdecke, Heusnerstrasse 40, 42283 Wuppertal, Germany; 9 Department of Pharmacology and Therapeutics, Faculty of Veterinary Medicine, Damanhour University, Damanhour, 22511 AlBeheira Egypt

**Keywords:** Drug repositioning, Emergence, Re-emergence, Viral agents, Drug resistance

## Abstract

The global impact of emerging and re-emerging viral agents during epidemics and pandemics leads to serious health and economic burdens. Among the major emerging or re-emerging viruses include SARS-CoV-2, Ebola virus (EBOV), Monkeypox virus (Mpox), Hepatitis viruses, Zika virus, Avian flu, Influenza virus, Chikungunya virus (CHIKV), Dengue fever virus (DENV), West Nile virus, Rhabdovirus, Sandfly fever virus, Crimean-Congo hemorrhagic fever (CCHF) virus, and Rift Valley fever virus (RVFV). A comprehensive literature search was performed to identify existing studies, clinical trials, and reviews that discuss drug repositioning strategies for the treatment of emerging and re-emerging viral infections using databases, such as PubMed, Scholar Google, Scopus, and Web of Science. By utilizing drug repositioning, pharmaceutical companies can take advantage of a cost-effective, accelerated, and effective strategy, which in turn leads to the discovery of innovative treatment options for patients. In light of antiviral drug resistance and the high costs of developing novel antivirals, drug repositioning holds great promise for more rapid substitution of approved drugs. Main repositioned drugs have included chloroquine, ivermectin, dexamethasone, Baricitinib, tocilizumab, Mab114 (Ebanga™), ZMapp (pharming), Artesunate, imiquimod, saquinavir, capmatinib, naldemedine, Trametinib, statins, celecoxib, naproxen, metformin, ruxolitinib, nitazoxanide, gemcitabine, Dorzolamide, Midodrine, Diltiazem, zinc acetate, suramin, 5-fluorouracil, quinine, minocycline, trifluoperazine, paracetamol, berbamine, Nifedipine, and chlorpromazine. This succinct review will delve into the topic of repositioned drugs that have been utilized to combat emerging and re-emerging viral pathogens.

## Introduction

Emerging and re-emerging viral agents are those viruses arising as a result of various changes, such as genetic alterations, drug resistance, climate change, and human movements, rendering them more infectious or virulent, or capable of infecting new hosts [1–3]. Some outstanding examples of emerging viral agents are the Ebola virus (EBOV), Zika virus (ZIKV), Human Immunodeficiency virus (HIV), Influenza virus, Monkeypox (mpox) virus, and SARS-CoV-2 (severe acute respiratory syndrome coronavirus-2) [2–5]. These viral pathogens pose significant threats to public health, and efforts are continuously undertaken to monitor and control their spread through surveillance, research, and development of vaccines and new therapies [5]. More importantly, the elevated spread, morbidity, and mortality rates of these viral pathogens can be partially attributed to the insufficient information available regarding their characteristics and virulence. For instance, EBOV caused 11,000 deaths during the 2014–2016 outbreak in West Africa [6]. The SARS-CoV-2 outbreak spanning from 2019 to 2023 led to the loss of millions of lives on a global scale [7, 8].

Several factors should be considered to effectively contain emerging viral pathogens. There is a lack of information available concerning virulence, transmission, and effective strategies for combating pathogens, especially those that are new or have been genetically modified [9]. A further concern is the rapid spread of these viral agents. Increased globalization, exemplified by heightened cross-border travel, commercial exchanges, and human migration, has been found to contribute to the rapid dissemination of novel viral agents within populations, posing challenges to their effective containment. It is imperative to invest significant financial and human resources into research, vaccine and therapy development, surveillance, and the implementation of control measures. Various limited-resources countries are struggling to stave off these diseases effectively. The issue of vaccine hesitancy should also be addressed. In many cases, effective vaccines are available to prevent the spread of the viral agents, but vaccine hesitancy and misinformation can lead to lower vaccination rates, making it difficult to achieve the desired levels of herd immunity and control diseases [1, 10–12]. It is noteworthy that political and social barriers are also a determining factor. While it is crucial for countries to cooperate and coordinate between themselves to eradicate emerging viral pathogens, political and social tensions may hinder access to essential information and resources. Indeed, global collaboration is vital to contain epidemics by ensuring effective prevention, detection, and control measures in the real world including an international partnership of governments, scientists, and healthcare workers [2, 11, 12].

**Fig. 1:**
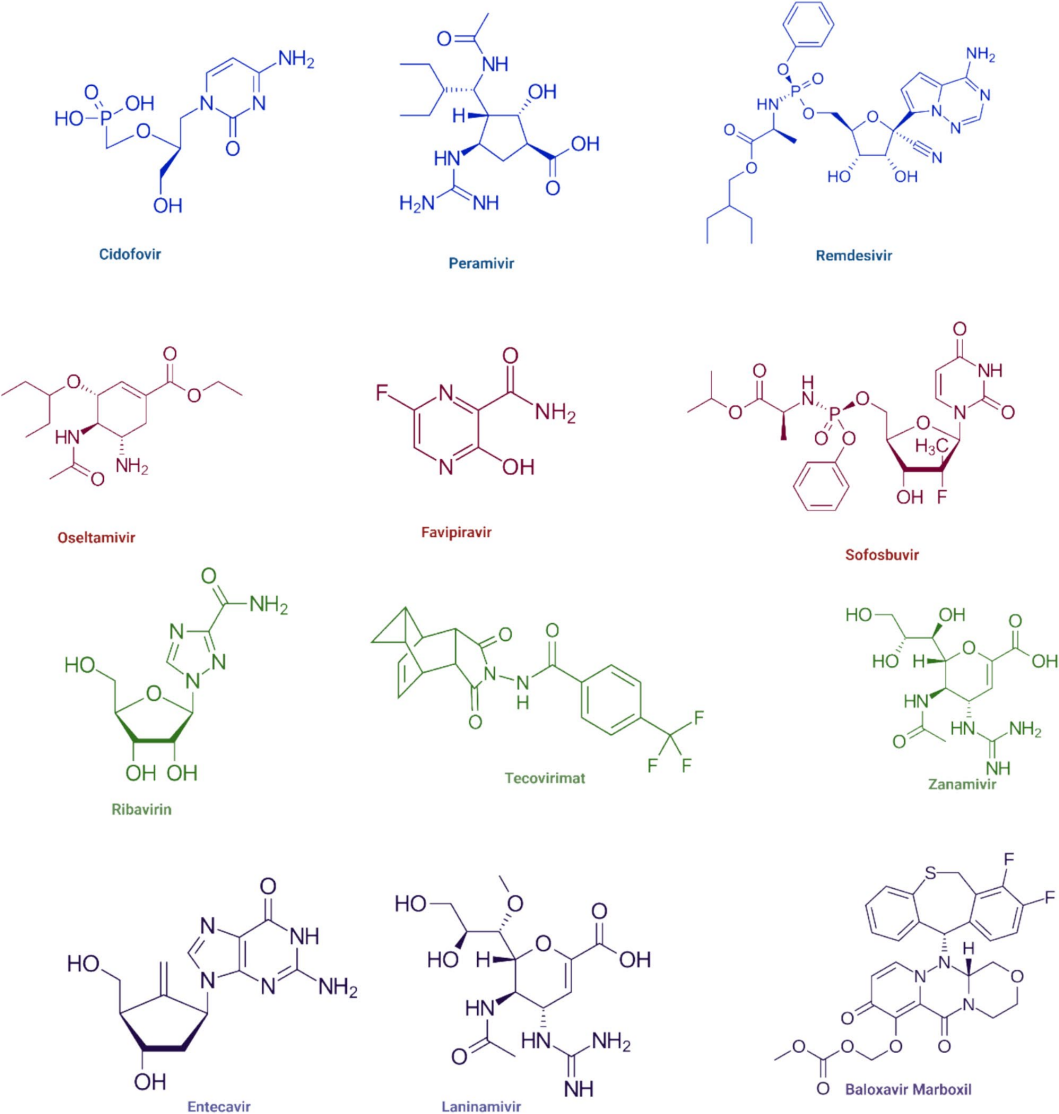
Structure of some antiviral drugs

**Fig. 2:**
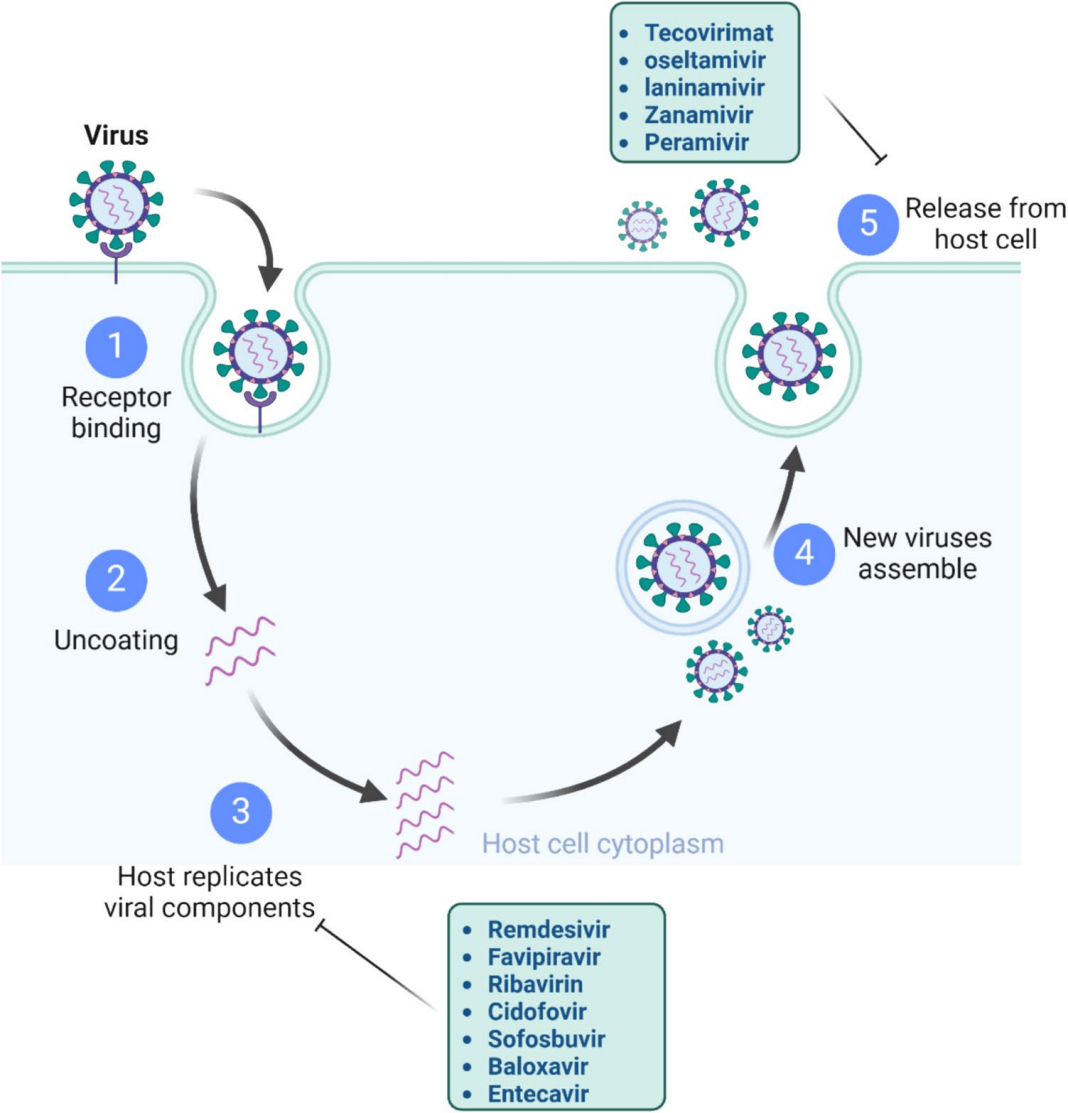
Sites or mechanisms of antiviral drugs action

Several antiviral drugs and their mechanisms have been included, such as remdesivir [13, 14], favipiravir [15, 16], ribavirin [17, 18], cidofovir [19], tecovirimat (ST-246) [20, 21], sofosbuvir [22, 23], oseltamivir (Tamiflu®) [24, 25], laninamivir [26, 27], zanamivir (Relenza®) [28], and peramivir (Rapivab®) [29], baloxavir marboxil, (xofluza®) [30] and entecavir [31], side effects and drug resistance has been reported [32–34] (Figs. 1 and 2).

As an anti-hepatitis C drug, remdesivir was subsequently investigated for EBOV and other viral hemorrhagic fevers. Drug-active metabolite precludes viral RNA polymerase. Remdesivir has shown broad spectrum antiviral properties against *Filoviruses*, *Coronaviruses*, *Metapneumoviruses*, *Paramyxoviruses*, *Flaviviruses*, *Arenaviruses*, *Bunyaviruses*, and *Togaviruses* both in vitro and in vivo [13]. Encouraging results have been observed in clinical trials regarding the use of the drug for managing COVID-19, particularly in patients with severe illness [35, 36]. Remdesivir has inferred reducing the recovery time of patients in clinical trials and has also been utilized in the treatment of SARS and Middle East respiratory syndrome coronavirus (MERS). The limitations of this drug involve its prodrug composition, subpar stability in vitro and in vivo scenarios, the prerequisite for hospitalization, exorbitant costs, lower recovery rates in certain research outcomes, and adverse effects including vomiting, nausea, and liver injury [13, 37, 38].

Additionally, there have been several combination regimens. For instance, sofosbuvir/velpatasvir regimen safety and efficacy was demonstrated after 12 weeks of administration against hepatitis C virus (HCV) genotype 4 [39]. Ledipasvir/sofosbuvir oral regimen has been approved against chronic HCV and in phase III trials demonstrated enhanced patients’ survival rate following 12 weeks of therapy [40]. It acts through inhibition of the viral genome replication in addition to anti-inflammatory effects. Among seven patients who received this combination treatment, three individuals experienced side effects, such as headache and oral thrush [41]. The combined administration of sofosbuvir and type-I interferons (α and β) has demonstrated synergistic properties, which have been implicated in exerting anti-Zika virus (ZIKV) effects in in vitro studies [42]. Tenofovir, an antiretroviral medication used to treat human immunodeficiency virus (HIV), has shown efficacy in combating hepatitis B by inhibiting the replication of its genome. The use of a combination therapy involving either tenofovir, alafenamide or tenofovir disoproxil fumarate with tenofovir alafenamide has yielded varying outcomes, indicating the necessity for additional research [43, 44]. Tenofovir alafenamide regimen was safe and precluded mother-to-child HBV transmission [45]. When IFN-β was combined with ribavirin or favipiravir, synergistic effects against rabies were observed. A drug cocktail involving ribavirin, Human rabies immune globulin (HRIG), mitogen-activated protein kinases (MAPKs) inhibitor, TNF-α inhibitor, caspase-1 inhibitor, interferon α/β and T-705 could significantly survive treated mice compared to the control [46].

The development of new drugs is a multifaceted endeavor that is impacted by factors, such as time, cost, and efficacy. This intricate process encompasses critical stages including research and discovery, preclinical testing, clinical trials, regulatory approval, manufacturing and scale-up, as well as marketing and distribution [47, 48]. Evidently, not all drug candidates complete the development process, and the costs incurred on failed projects contribute to the overall expenses. Complicating the matter further, the time required for drug development can span over a decade or more [49, 50]. A drug repositioning strategy involves using an existing drug to treat a different illness. As many of these drugs already have safety and efficacy data, this approach can be a cost-effective and time-saving way to achieve novel choices. There are diverse strategies for repositioning drugs, such as investigating their pharmacological properties to identify targets, screening of existing drugs against disease-specific cells or animal models or using computational approaches to predict their potential effectiveness [47, 48]. Thus far, several repositioned drugs have demonstrated potential antiviral properties through diverse mechanisms. The present review has focused upon the current status of drug repositioning in the battle against newly emerging and re-emerging viral diseases. The selection of viral diseases for inclusion in this review is influenced by the priorities set forth by the Coalition for Epidemic Preparedness Innovations (CEPI) foundation. Nevertheless, other viral diseases falling outside of these criteria may also be part of the current review [51].

## SARS-COV-2

With coronavirus disease 2019 (COVID-19) deaths on the rise, researchers are studying ways to repurpose already-approved drugs to combat Severe Acute Respiratory Syndrome Coronavirus 2 (SARS-CoV-2). Dexamethasone is a synthetic glucocorticoid with anti-inflammatory and immunosuppressant properties to treat a broad spectrum of inflammatory illnesses. There is evidence to suggest that the drug can reduce mortality in severely ill COVID-19 patients, especially when given in minimal dosages [52]. Non-severe patients may also experience potential side effects from corticosteroids [53]. Among corticosteroids, dexamethasone has broader clinical application with lower adverse effects [54], which mainly occur following long-term use as forms of weakness, weight gain, edema, and necrosis [55].

Baricitinib is a first generation of Jakinibs (Janus tyrosine kinase (JAK) inhibitors) utilized for the treatment of several inflammatory diseases, such as rheumatoid arthritis and alopecia areata. It also enhances SpO2/FiO2 (peripheral capillary oxygen saturation /fraction of inspired oxygen) ratio [56]. Studies have revealed that the drug is effective in blocking viral entry and decreasing inflammatory markers in COVID-19 patients. Its use in combination with remdesivir is currently under scrutiny [57, 58].

Tocilizumab, an IL-6 receptor antagonist, is a monoclonal antibody that has been humanized to act as an immunosuppressant. It is utilized in the treatment of idiopathic arthritis and rheumatoid arthritis [59, 60]. When administered to patients with severe COVID-19, the drug reduced mortality rates [15]. In COVID-19 patients, significant positive outcomes have been inferred in relation to outcome measures, such as intensive care unit (ICU) admission, mortality, and mechanical ventilation, but not for those with mild disease or prolonged invasive mechanical ventilation [61]. Apart from tocilizumab, several anti-cytokine/chemokine monoclonal antibodies are being evaluated in clinical trials for the management of COVID-19 patients. Notable examples include lenzilumab (a GM-CSF antagonist), risankizumab (humanized monoclonal antibody against interleukin-23), gimsilumab (KIN-190 N), mavrilimumab/KPL 30D, TJ003234 (three other anti-GM- CSF monoclonal antibodies), leronlimab (CCR5 antagonist), canakinumab (Anti IL-1β), CPT-006 and AK119 (Anti CD73), garadacimab/CSL312 (Factor XIIa antagonist), pamrevlumab (monoclonal antibody against connective tissue growth factor), bevacizumab (Anti VEGF), cizanlizumab (Anti P-selectin), ravulizumab (Anti C5), emapalumab (IFNɣ antagonist), and anakinra (IL-1 antagonist) [62].

Thus far, several humans, humanized, or bioengineered monoclonal antibodies designed for therapeutic purposes, which target various regions of the S-protein of SARS-CoV-2, have received emergency use authorization (EUA) following the analysis of Phase 1/2 and Phase 2 data. These monoclonal antibodies (e.g., CR3002, F26G19, 2B2, 1A9, 4B12, 1G10, and S309) can interact with SARS-CoV-2 by binding to the receptor-binding domain (RBD) [63]. REGN-COV2 emerges as one of the first monoclonal antibody cocktails sanctioned for the treatment of SARS-CoV-2 infection. This therapeutic combination features two potent neutralizing IgG1 monoclonal antibodies, casirivimab and imdevimab, each with unmodified Fc regions. These antibodies are strategically designed to bind to distinct epitopes within the RBD of the virus [64]. The two anti-spike candidate medications currently in Phase 3 clinical trials are the Eli Lilly combination of LY3819253 (LY-CoV555) with LY3832479 (LY-CoV016) and VIR-7831/GSK4182136 (Sotrovimab). Another combination therapy, authorized for emergency use, is the AbCellera Biologics and Eli Lilly Bamlanivimab, which consists of bamlanivimab and etesevimab, and is administered exclusively to newly diagnosed mild/moderate COVID-19 patients [62].

The anti-parasitic activity of Ivermectin is manifested through the paralysis and eventual death of parasites, achieved by its binding to glutamate-activated chloride channels [65]. Ivermectin may also exert antiviral properties via inhibition of SARS-CoV-2 proteins nuclear import by binding to and destabilizing IMPα/β1, like observations made in other RNA viruses [66, 67]. Nevertheless, additional clinical studies are required prior to endorsing the drug as a standard treatment option [68].

## Ebola virus

Ebola virus disease (EBOVD), also known as Ebola hemorrhagic fever, mainly occurring in Central and West Africa, is a severe and often fatal illness in humans caused by the EBOV [69]. The virus is transmitted to humans from wild animals, such as chimpanzees, fruit bats, and gorillas, and can then be spread through human-to-human transmission via direct contact with bodily fluids of infected individuals or through contact with contaminated objects or surfaces [70, 71]. The typical symptoms of the disease encompass headache, fever, myalgia, fatigue, vomiting, diarrhea, abdominal pain, weakness, and unexplained bruising or bleeding [70–72]. Patients often experience an incubation period of 2–21 days, with rapid onset of severe symptoms that demand hospitalization. There is currently no approved treatment for EBOVD. Supportive care, such as fluid balance maintenance, replacing lost blood, and treating secondary infections could enhance survival rates [70, 71]. Prevention measures entail avoiding contact with infected individuals or their bodily fluids, practicing good hygiene, and implementing infection control protocols in healthcare settings [73]. Drugs toremifene and ibuprofen could bind to a cavity between EBOV glycoproteins of attachment and fusion subunits (GP1 and GP2) and destabilize its structure in an in silico study [74].

ZMapp is a novel biopharmaceutical medication consisting of three chimeric monoclonal antibodies (c2G4, c4G7, and c13C6) against EBOV glycoprotein that is currently being researched as a potential therapy for EBOV [75]. Additionally, ZMapp strengthens the respiratory mucosal defenses in fighting EBOV by limiting the mobility of Ebola pseudo virus within the mucus secretions [76]. Despite lacking approval for widespread utilization, the drug has exhibited promising results in clinical trials [75]. According to a new systematic review, ZMapp Plus has been identified as the most effective treatment for EBOV when compared to the current standard of care and the other two drugs included in the analysis [77]. Among the other glycoprotein-targeting monoclonal antibodies (mAb), MIL77E, mAb114, and REGN-EB3 have proven to be highly effective. The possibility of combining monoclonal antibodies into a cocktail represents a promising avenue that warrants further exploration and exploitation. However, to rationally design successful mAb cocktails, it is essential to identify the precise epitope recognized by different monoclonal antibodies through genetics and/or structural biology. Additionally, new epitopes and key residues of the above-mentioned glycoprotein, such as Q206 and Q411, which have been identified as targets for new potential candidate mAbs for EBOV prevention and treatment, should be taken into consideration [62].

Artesunate, a semi-synthetic compound derived from artemisinin, is employed in the management of malaria infections ranging from mild to severe. In recent years, promising results have been observed in utilizing artesunate for its antiviral properties against EBOV [78]. The findings of a recent retrospective cohort study indicated that administering early oral antimalarial treatment during an EBOV outbreak was associated with a reduction in mortality rates. However, further research is necessary to better understand the effects of this treatment on patients infected with EBOV [79]. Another study indicated that the risk of death was lowered by 31% in a group of 71 patients who were treated with artesunate-amodiaquine as opposed to those who received artemether-lumefantrine [78]. Amodiaquine, which shares structural similarity with chloroquine, is a commonly used antimalarial medication in Africa with unproven antiviral effects [80]. It exhibits antiviral activity against various stages of viral life cycles of HIV-1 and coronaviruses. The potential activity of the drug against EBOVD could be achieved by prohibiting viral entry into host cells. While certain research indicates positive findings in animal models, there have been no clinical trials carried out for chloroquine in the treatment of EBOVD thus far [81].

## Herpes simplex virus

The Herpes simplex virus (HSV) is a common viral infection causing sores or blisters on various areas of the body. Two distinct types of HSV exist, with HSV-1 leading to oral herpes (cold sores) and HSV-2 resulting in genital herpes. The virus is spread through direct contact with infected patients in genital route or via skin, saliva, or genital secretions [82]. Symptoms may include painful blisters or sores, itching or burning sensations, and flu-like symptoms, such as fever, headache, and swollen glands.

While there is no cure for HSV, antiviral medications can help reduce the severity and frequency of outbreaks and lower the risk of transmission to others. HSV patients should avoid sexual contact during outbreaks and inform sexual partners of their status [83]. Acyclovir is the medication of choice for treating HSV, as well as other infections that are similar in nature. Acyclovir acts via inhibiting viral DNA replication and is effective against both HSV-1 and HSV-2. Despite this, there has been an emergence of acyclovir-resistant strains of HSV, prompting researchers to explore alternative drugs that could be effective against these resistant strains [84].

Valproic acid, a drug commonly used to treat epilepsy and bipolar disorder, was among the pharmaceutical options being explored for its efficacy in combating HSV infections. Valproic acid has been shown to impede HSV replication and to reduce the frequency of recurrent HSV infections [85]. Valproic acid can also downregulate viral gene expression [86]. Other candidates for drug repositioning against HSV infections include chloroquine and imiquimod (a drug for treating genital warts, superficial basal cell carcinoma, and actinic keratosis) [87]. However, they may predispose patients to develop herpes zoster or dermatomyositis/polymyositis [88].

## Monkeypox (Mpox) virus

Mpox is caused by a DNA virus in the family *Poxviridae* and Orthopoxvirus genera, primarily occurring in remote parts of Central and West Africa, North and South America and Europe [89]. The symptoms of Mpox are relatively mild, mainly including fever, headache, myalgia, lymph nodes swelling, chills, and exhaustion, followed by rash in various body parts. A rash then develops, often beginning on the face and then spreading to other body parts. Mpox is usually a self-limited disease, with most cases resolving within 2–4 weeks [90]. However, severe cases can occur, particularly among immunosuppressed individuals. The virus is mainly transmitted from human-to-human (sexually, respiratory droplets, bodily fluids) or transmission through respiratory droplets, bodily fluids, or contaminated materials [91]. There is no approved antiviral drug, but supportive care can be provided to reduce symptoms. Preventive measures include avoiding contact with infected animals, sufficient hand hygiene, and use of personal protective equipment (PPE) when handling animals or caring for infected individuals. There should be an emphasis on repurposing drugs against Mpox. Those vital viral proteins and enzymes, such as kinases and phosphatases include crucial targets [92]. Molecular dynamics simulation analyses have discovered three FDA-approved drugs of saquinavir, capmatinib, and naldemedine which simultaneously target p37, the viral topoisomerase, and thymidylate kinase [93].

## Influenza virus

Influenza viruses are a group of RNA viruses in the family *Orthomyxoviridae*, causing a respiratory illness with a range from mild to severe symptoms or even fatal cases. There are four types of influenza viruses, including A, B, C, and D. Influenza A and B are highly contagious viruses responsible for most seasonal flu outbreaks. Influenza C causes milder respiratory symptoms [94]. Influenza A viruses are categorized into different subtypes according to the surface proteins hemagglutinin (H) and neuraminidase (N), as they evolve through mutations and give rise to new viral strains. Thus, new influenza vaccines are developed every year to tackle the most recent circulating strain [95]. Therefore, WHO issues recommendation for formulation of flu vaccines annually for Northern and Southern hemisphere in view of circulating strains [95].

Several pharmaceuticals have exhibited anti-influenza activity, suggesting potential for drug repositioning. In this respect, anticancer drugs (gemcitabine and trametinib), statins (cholesterol modulators), nitazoxanide (anti-parasitic drugs), peroxisome proliferator activator receptor (PPAR) antagonists (anti-hyperlipidemic drugs), celecoxib and naproxen (COX-2 inhibitors), ruxolitinib (approved for myelofibrosis treatment), dapivirine (phase III anti-HIV drug), metformin (approved type 2 diabetes drug), lisinopril (anti-hypertensive drug), dorzolamide (approved anti-glaucoma drug), midodrine (approved anti-hypotensive drug), and diltiazem (approved anti-hypertensive drug) are prominent examples [96].

Avian flu, also known as bird flu, is caused by influenza viruses subtypes A (H10N3, H5N6, H5N1, H7N9, and H9N2) naturally occurring in birds including wild birds and domesticated poultry, such as chickens, ducks, and turkeys. The disease is rare in humans [97, 98]. Symptoms may range from mild to severe including fever, cough, sore throat, myalgia, and eye infections. There is no approved drug against the virus. Drug repositioning has evaluated several existing drugs as therapeutic potential against avian flu. Among them, amantadine which is an inhibitor of M2 protein [99].

The human immunoglobulin G1 (IgG1) monoclonal antibody MHAA4549A specifically binds to a conserved epitope located on the stalk of influenza A hemagglutinin (HA), leading to the neutralization of all known human influenza A strains [100]. Extensive evaluation in Phase 1 and Phase 2 clinical trials has confirmed the efficacy and safety of MHAA4549A. Notably, this antibody has exhibited a favorable safety profile in healthy individuals [101]. Broadly neutralizing antibodies targeting influenza viruses could also serve as a promising strategy for both the prevention and treatment of influenza. Among the earliest monoclonal antibodies with the capacity for broad neutralization is C179, which demonstrates effectiveness against the H5, H6, and H9 strains of the influenza virus [102]. CR9114 is another example of broadly neutralizing antibodies that demonstrates potent inhibitory activity against both influenza A and B viruses [103]. CR9114 is recognized as one of the most effective broadly neutralizing antibodies against multiple subtypes of influenza A virus, outperforming other notable antibodies, such as CR6261, F10, 12D1, CR8020, and FI615 [102].

## Hepatitis viruses

Hepatitis can be caused by several viruses including hepatitis B and C viruses (HBV and HCV, respectively). There are several drugs repurposed against hepatitis viruses. Interferons, as a part of innate immunity, participate in antiviral immune responses. They have been employed against HBV and HCV for decades, and work via the immune system provocation to combat the viruses [104]. Hepatitis A and E viruses (HAV and HEV, respectively) affecting the liver, appear as acute hepatitis among children and adults [105].

Currently, there are no approved antiviral medications available for the eradication of HAV or HEV. Nonetheless, drug repositioning presents a promising strategy for developing new therapeutic options. Preclinical evidence suggests that ribavirin may be effective against HAV and HEV in cell culture and animal models. For the majority of transplant recipients, interferon-α is not a suitable option, although it is being considered as a potential drug candidate for hepatitis A and E viruses [106]. The activity of interferon-α against HAV and HEV has been observed in cell culture and animal experiments. It is worth noting that gemcitabine, a medication used in cancer treatment, has shown significant anti-HEV activity against genotypes 1 and 3 by inducing STAT1 phosphorylation and eliciting interferon-like responses in cell culture [107]. Moreover, it is of interest to mention that artesunate displayed inhibitory effects against HEV-1 and HEV-3 genotypes through the suppression of viral RNA polymerase and helicase in vitro [108].

## Chikungunya virus

Chikungunya virus (CHIKV) is classified under the *Alphavirus* genus within the *Togaviridae* family, characterized as a single-stranded RNA virus. The virus has worldwide spread mainly locating in North and South America, Africa and Southeast Asia [109]. The virus is transmitted to humans by the bite of infected *Aedes* mosquitoes, primarily *Aedes aegypti* (*A. aegypti)* and *A. albopictus* [110]. The virus causes CHIK fever, a disease characterized by fever, joint pain, myalgia, headache, nausea, fatigue, and rash. Symptoms may arise 3–7 days after being bitten by an infected mosquito and can endure for several weeks [111]. Even though the disease is not often deadly, it typically results in debilitating outcomes, including prolonged joint pain. Given the current lack of an approved treatment or vaccine for CHIKV, the reduction of morbidity rate relies on the implementation of mosquito control measures, personal protective strategies, and the use of repellents [112]. Notably, drug repositioning is a promising strategy to identify new treatment options. For instance, chloroquine by modifying the intracellular pH level and blocking viral replication in both cell culture and in vivo studies, serves as an additional repurposed anti-CHIKV drug [113]. Zinc acetate, an FDA-approved drug, is an important compound that has demonstrated inhibitory effects against CHIKV nsp-2 in both in silico and in vitro experiments [114].

## Zika virus

The Zika virus (ZIKV), initially discovered in the Zika Forest of Uganda in 1947, is a viral pathogen transmitted by mosquitoes, particularly *Aedes mosquitoes*. This flavivirus is known to result in significant birth defects in newborns born to mothers who have been infected [115]. The virus has been reported in many countries, particularly in Central and South America, the Caribbean, and Southeast Asia [116]. Most individuals do not exhibit any symptoms or only experience mild symptoms, typically characterized by fever, rash, joint pain, myalgia, and headache. Nevertheless, if a pregnant woman contracts ZIKV, it can lead to severe congenital abnormalities like microcephaly in the unborn child [3]. There is presently no authorized treatment or vaccine for ZIKV infection, although supportive care can help relieve symptoms. Pregnant women should take extra precautions to avoid mosquito bites and consult with their healthcare provider if traveling to areas with ZIKV epidemics.

Several drugs have been repurposed to target ZIKV. For example, chloroquine has shown antiviral properties against ZIKV in both in vitro (human neural stem, brain microvascular endothelial, and Vero cells) and in vivo (mouse neurospheres) studies [117]. It further prevented the vertical transmission of the virus by impeding the release of viral RNA from endosomes [118]. Dosage optimization involved administering 600 mg on the first day, 300 mg on the second day, and maintaining a daily dose of 150 mg thereafter [119]. In addition, favipiravir has displayed antiviral effects against ZIKV [120]. The combination of favipiravir and ribavirin reduced cell death and prohibited ZIKV replication in various human cancer cells [121].

Ivermectin, a medication used to treat parasitic infections, has demonstrated varying levels of effectiveness against the Zika virus in both in vitro and in vivo studies. Nevertheless, ivermectin did not exhibit any antiviral effect in IFNAR1 knockout mice [122]. Suramin, yet another anti-parasitic drug, has shown potential anti-ZIKV properties through its interference with virus-host cell binding/entry, reduction of genome replication, and inhibition of ZIKV release [123]. Suramin effectively suppressed viral replication by interacting with NS3 helicase and decreasing viral plaque forming units (PFU) by 3–5 log10 at concentrations ranging from 2.5 to 5µg/mL [124]. By utilizing Mycophenolic acid (MPA) and 5-fluorouracil at concentrations of 2 and 0.5 µM, C6/36 cells were protected from ZIKV infection as viral replication was inhibited [124].

Monoclonal antibody DMAb-ZK190, specifically created for Zika, targets the ZIKVE protein on the Zika virus. Studies conducted in vivo have shown that synthetic antibody rapidly protects mice and monkeys from Zika and may control infection. Clinical trials are currently underway for this promising monoclonal antibody [125]. Another research revealed that the use of two antibodies, Z004 and Z021, on pregnant macaques shields the fetus from neurological impairment and decreases vertical transmission of the Zika virus [126].

## Dengue fever virus

Dengue fever virus (DENV) is a virus spread by mosquitoes, particularly *Aedes mosquitoes*. It is classified under the *Flavivirus* genus and *Flaviviridae* family. This virus leads to dengue fever, which is characterized by symptoms including high fever, severe headache, muscle and joint pain, rash, and sometimes bleeding [127]. Annually, 100 million individuals worldwide are affected by the virus mainly occurring in North and South America, Europe and East and Southeast Asia [128]. Other routes of transmission include blood transfusion, organ transplantation, or from mother to neonates during childbirth. The virus has four serotypes (DEN-1, DEN-2, DEN-3, and DEN-4). The absence of an approved antiviral drug means that supportive care, including rest, hydration, and pain relief, is the most effective treatment. Owing to the limited availability of approved antiviral medications for DENV eradication, drug repositioning has shown promise in introducing new antiviral agents.

A drug showing potential in preclinical trials is chloroquine, which has been repurposed for treating autoimmune conditions, such as lupus and rheumatoid arthritis. This compound reduces intracellular pH level, resulting in viral replication inhibition. Chloroquine has demonstrated antiviral activity against DENV serotype-2 in preclinical studies, as shown in vitro (Vero at 50 μg/mL, U937, plasmacytoid dendritic cells) [129, 130] and in vivo [131].

Lovastatin, a medication often prescribed to reduce cholesterol, is being explored as a potential drug for combating DENV. Lovastatin has demonstrated inhibitory effects on the replication of DENV in both in vitro (human peripheral blood mononuclear, dermal microvascular endothelial, HMEC-1, Vero, and monkey kidney cells) [132, 133] and in vivo (AG129 mice, 200 mg/kg/day) studies [134] through reducing the lipid biosynthesis rate, required for the viral replication. Other prominent repurposed drugs against DENV include ivermectin [135], nitazoxanide [136], quinine [137] and minocycline [138], trifluoperazine [139]and *N*-acetyl cysteine [140]. Moreover, a comprehensive omics and network analysis identified valproic acid, resveratrol, and paracetamol as effective anti-DENV agents [141].

## West Nile virus

West Nile virus (WNV) is a flavivirus that is primarily transmitted to humans, birds, and horses through the bites of infected mosquitoes. Infections by the virus target the central nervous system and meninges, resulting in mild febrile illness. Local outbreaks have been observed in Europe, America, Africa, and Asia [142]. While there are no approved antiviral drugs available for the virus, drug repositioning presents a hopeful strategy for identifying novel therapeutic options. By employing various omics and network projection analyses, researchers identified nucleophosmin (NPM1) as a potential drug target that is commonly found in mosquito-borne viruses including ZIKV and WNV [141].

## Japanese encephalitis virus (JEV)

Japanese encephalitis virus (JEV), a flavivirus, is the primary cause of encephalitis in the Asia–Pacific region with the potential for global transmission through the bites of *Culex* species and Anopheles *plumbeus* mosquitoes [143]. Recently, the emergence of genotype IV (GIV) in Australia has led to 42 reported cases and seven fatalities [144]. The results of a network analysis in an in silico study demonstrated that tanespimycin, staurosporine, LY-294002, dinoprostone, alvespimycin, and acetylsalicylic acid were identified as repurposed drugs effective against both JEV and DENV [141]. Berbamine decreases the low-density lipoprotein receptor (LDLR) as the viral receptor at plasma membrane and thus mitigates viral entry [145]. Accurate drug delivery systems and combination therapies are required against the virus [146].

## Rabies

Rabies is caused by a negative-sense RNA and enveloped virus known as Rhabdovirus affecting the nervous system and transmitted to humans through the bite and saliva of infected animals [147]. There are currently no approved antiviral drugs to combat the virus, but drug repositioning is a promising strategy to discover novel treatment options. Ribavirin and INF-α have outlined antiviral effects in vitro and in vivo [148]. Also, another study demonstrated that combination of IFN-β and sorafenib inhibited relative viral replication by 77.19%.

## Sandfly fever and severe fever with thrombocytopenia syndrome viruses

Sandfly fever is a viral disease belonging to RNA viruses of family *Phenuiviridae*, order *Bunyavirales* and transmitted to humans through the bite of infected sandflies. The disease symptoms mainly include severe fever and nervous system infection [149]. There are currently no approved antiviral drugs against the virus.

Severe fever with thrombocytopenia syndrome virus (SFTSV) is a novel bunyavirus causing high rate of death particularly in Eastern Asian countries with recent increasing trend. Various wild and domestic animals play a role as reservoirs [150]. The disease fatality rate has included 2.8–48% in various global areas [151]. As a priority of research by the world health organization (WHO), there is no approved antiviral drug [151]. The treatment with bortezomib (interfere with viral replication and release in vivo) [152], nifedipine, benidipine hydrochloride (in vitro and in vivo) [153], and hexachlorophene (IC_50_: 1.3 ± 0.3 mM) interfering with cell membrane fusion [154]. A mAb known as Ab10 binding to Korean SFTSV strains recombinant Gn glycoprotein has also inferred antiviral effects [155].

## Rift Valley fever

Rift Valley fever (RVF) is a viral disease that affects both humans and animals. RVF virus (RVFV) is a single-stranded RNA virus belonging to the genus *Phlebovirus* and family *Bunyaviridae*. It is primarily transmitted to humans through contact with the blood, bodily fluids, or tissues of infected animals, particularly livestock, such as sheep, goats, and cattle [156]. Humans can also become infected following bite of infected mosquitoes. There are currently no approved antiviral drugs approved for RVFV, but drug repositioning is a promising strategy to develop new therapeutic options. Potential repurposed drugs have included suramin [157].

## Crimean-Congo hemorrhagic fever virus

An RNA virus called Nairovirus is responsible for Crimean-Congo hemorrhagic fever (CCHF), and it is transmitted to humans through the bite of infected ticks or by contact with blood or tissues from infected animals or humans [158]. The transmission of the disease has been intensified by climate change and globalization [159]. Currently, there are no authorized antiviral drugs available for treating the CCHF virus. Nonetheless, drug repositioning stands out as a promising strategy for uncovering new treatment options. In this regard, chloroquine and chlorpromazine demonstrated the ability to inhibit viral multiplication when tested in vitro with Vero E6 and Huh7 cells [160].

In Table 1, a summary of main drugs repurposed for combating emerging and re-emerging viral agents are listed.

**Table 1:** Main drugs repurposed against emerging and re-emerging viral agents and their mechanisms of action

Drug	Mechanism of action	Agents	
Chloroquine	Inhibition of attachment and entry, proteases, modifying enzymes and viral budding, alteration of the intracellular pH level and inhibiting viral replication, inhibition of virus vertical transmission via hinder of endosomal viral RNA release	SARS-CoV-2 (CT-III), HSV (PCS), CHIKV (CT-III)^Ter^, ZIKV (PCS), EBOV (PCS), DENV (CT-II), CCHFV (PCS), HIV-1 (CT-II)	
		SARS-CoV-2 (CT-IV), DENV (CT-II), ZIKV (PCS)	
Ivermectin	Inhibition of nuclear import of viral proteins	SARS-CoV-2 (CT-IV), DENV (CT-II)	
Dexamethasone	Binding to glutamate-activated chloride channels, inhibition of viral proteins nuclear import by IMPα/β1		(CT-III)
Baricitinib	JAK inhibition, enhancement of SpO_2_/FiO_2_ ratio, inhibition of viral entry, reduce of inflammatory markers	SARS-CoV-2	(CT-II)
Tocilizumab	IL-6 receptor antagonist		(CT-III)
Mab114(Ebanga™)	Targeting EBOV glycoprotein (GP), specifically its receptor-binding domain (RBD) and neutralizing the virus and inhibiting infectivity [161]		(CT-III)
REGN-EB3	Targets EBOV glycoprotein using three fully human monoclonal antibodies: atoltivimab, maftivimab, and odesivimab [162]	EBOV	(EA)
ZMapp (pharming)	Mixture of three monoclonal antibodies directed against the surface glycoprotein of EBOV; reinforces respiratory mucosal barriers through reducing penetration		(CT-III)
Artesunate	Inhibition of viral RNA polymerase and helicase	EBOV (CT-III)^Ter^, HEV-1 (PCS), HEV-3 (PCS)	
Valproic acid	Impeding viral replication and downregulating the genes expression	HSV-1	(PCS)
Imiquimod	Suppression of virus by upregulating cystatin A via adenosine receptor A1 pathway, independently of TLR7 and IFNs, inhibiting viral propagation in non-immune cells [163]		(CT-III)
Saquinavir	Targeting p37, viral topoisomerase and thymidylate kinase		(PCS)
Capmatinib		Mpox	(PCS)
Naldemedine			(PCS)
Trametinib	Blockade of viral replication of IAV subtypes and interfering with export of progeny vRNPs from the nucleus [164]		(PCS)
Statins	Inhibition of the Rho/Rho kinase pathway, Reduction of proinflammatory cytokines and chemokines, Blockade of downstream molecules key to virus infectivity, Attenuation of proinflammatory cytokine response [165]. Atorvastatin and lisinopril inhibit influenza virus replication by restricting lipid droplet formation, decreasing neutrophil influx and changing nitric oxide balance [166]	Influenza virus	(CT-II)
Celecoxib	Reduces inflammation and mortality in influenza viruses by attenuating TNF-α, G-CSF, and IL-6 levels, suggesting an anti-inflammatory mechanism of action [167]		(CT-III)
Naproxen	Inhibits Influenza A virus by binding to the nucleoprotein’s RNA groove, disrupting NP-RNA association crucial for viral replication [168]		(CT)^Ter^
Metformin	Inhibition of viral replication and influenza A virus-induced cytokine expression, reduction of MCP-1 and IP-10, cytokines [169–171]		(CT-I)
Ruxolitinib	Anti-inflammatory and immunosuppressive effects and inhibition of viral replication via modulating the cellular signaling cascades[169, 171]	Influenza virus (PCS), HIV-1 (CT-II) [169]	
Nitazoxanide	Inhibition of Influenza virus by blocking maturation of viral hemagglutinin and DENV virus by inhibiting viral transcription factor IE2, antiviral activity through eukaryotic translation initiation factor 2α [172]	Influenza virus (CT-III), DENV (PCS)	
Gemcitabine	STAT1 phosphorylation and elicit of interferon-like responses (K. [173])	Rotavirus (PCS), MERS-CoV (PCS), Influenza virus (PCS), HEV genotype 1 & 3 (PCS)	
Dorzolamide	(anti-glaucoma drug)		(PCS)
Midodrine	(anti-hypotensive drug)	Influenza virus	(CT-II)
Diltiazem	(anti-hypertensive drug)		(CT-II)^Ter^
Amantadine	Inhibitor of M2 protein	Influenza viruses subtypes A (H10N3, H5N6, H5N1, H7N9, and H9N2) (CT-II)	
Zinc acetate	Inferred nsp-2 inhibitory effects	CHIKV (PCS)	
Suramin	Interfering in the virus-host cell binding/entry, reduction of genome replication and virus release, inhibited viral replication via binding to NS3 helicase and reducing 3–5 log10 of viral plaque forming unit (PFU) at 2.5–5 µg/mL, Inhibiting the replication by disrupting nucleocapsid-RNA interactions [157]	CHIKV (PCS), Ebola (PCS), and ZIKV (PCS), RVF (PCS)	
5-fluorouracil	Inhibition of viral replication	ZIKV (PCS)	
Lovastatin (a statin)	Inhibit of replication; reducing the lipid biosynthesis rate, required for the viral replication	DENV (PCS)	
Quinine	Significantly inhibiting DENV replication by reducing DENV RNA and viral protein synthesis in a dose-dependent manner [137]	HSV-1 (PCS), SARS-CoV (PCS), DENV (PCS)	
Minocycline	Reduces DENV viral RNA synthesis, intracellular envelope protein expression, and infectious virion production, while upregulating the transcription of antiviral genes [138]	HIV (PCS), SARS-Cov-2 (PCS), DENV (PCS)	
Trifluoperazine	The Food and Drug Administration-approved antipsychotic drug trifluoperazine, a calmadulin antagonist, inhibits viral replication through PERK-eIF2α axis [174, 175]	SARS-Cov-2 (PCS), ZIKV (PCS), DENV (PCS)	
Valproic acid	Interacting with E protein of other flaviviruses [176]	Flaviviruses (PCS)	
Resveratrol	Impacting on the viral protein expression, RNA translation, and replication [177]	DENV	
Paracetamol	Pain relief	Influenza virus (CT-IV), DENV (CT-II) [178]	
Berbamine	Decreases the low-density lipoprotein receptor (LDLR) as the viral receptor at plasma membrane and thus mitigates viral entry	SARS-COV-2 (CT-IV), ASFV (PCS), JEV (PCS), DENV (PCS)	
INF-β	Inhibits viral replication	Rhabdovirus (PCS)	
Drug cocktail (Ribavirin, HRIG, MAPKs inhibitor, TNF-α inhibitor, caspase-1 inhibitor, interferon α/β, T-705)	Targeting the viral replication at different stages of the virus life cycle and inhibiting some pathways of the innate host immune response [46]		
Bortezomib	Interfering with viral replication and release, Blockade of early steps infection by a proteasome inhibitor [179]	HSV (PCS), CHIKV (PCS), SFTSV (PCS)	
Nifedipine	Targeting the host calcium channels and viroporins: a promising strategy for SARS-CoV-2 and Severe Fever With Thrombocytopenia Syndrome Virus Infection therapy [180]	SARS-CoV-2 (CT-IV), SFTSV (PCS)	
Benidipine hydrochloride	Interfering with cell membrane fusion	SFTSV (PCS)	
Ab10	Binds to recombinant Gn glycoprotein	Korean SFTSV strains (PCS)	
Chlorpromazine	Inhibition of viral multiplication	CCHF (PCS)	

*SARS-CoV-2* Severe acute respiratory syndrome coronavirus 2, *HSV* herpes simplex virus, *CHIKV* chikungunya virus, *ZIKV* Zika virus, *EBOV* Ebola virus, *DENV* dengue virus, *CCHFV* Crimean Congo hemorrhagic fever virus, *RVF* Rift valley fever, *HIV* human immunodeficiency virus, *HEV* Hepatitis E virus, *mpox* monkeypox, *MERS-CoV* Middle East Respiratory Syndrome Coronavirus, *JEV* Japanese encephalitis virus, *WNV* West Nile virus, *SFTSV* Severe fever with thrombocytopenia syndrome virus, *CT-I* clinical trial phase 1, *CT-II* clinical trial phase 2, *CT-III* clinical trial phase 3, *CT-IV* clinical trial phase 4, *EA* expanded access, *PCS* pre-clinical studies (in vitro and in vivo studies); ^Ter^: Terminated

## Future directions and conclusion

The practice of therapeutic switching, drug repurposing, or drug repositioning brings about several benefits, including reduced time and costs, increased success rates, expanded patient populations, improved safety, rapid translation to clinical practice, and the leveraging of existing knowledge. The spread of emerging or re-emerging viral diseases is influenced by the intricate interplay of global population growth, urbanization, international travel and trade, and climate change. Addressing the challenge of such threats necessitates a multifaceted plan that includes public health interventions, research endeavors, and the development of novel vaccines and antiviral treatments. Surveillance plays a crucial role in controlling and preventing the dissemination of these viruses by enabling early detection and monitoring of potential outbreaks. Regular screening of individuals, animals, and vectors, including mosquitoes or ticks, is of paramount importance. The implementation of public health measures, such as quarantine, isolation, and social distancing plays a significant role in minimizing the spread of the virus. Advancements in drug repositioning or accelerating the discovery of viruses in the future may be driven by several key factors, such as computational approaches, viral biology insights, collaborative research, drug combination screening, phenotypic screening and performance of clinical trials [181–183]. Machine learning and artificial intelligence are crucial in prompt and accurate drug screening and discovery via identifying potential drug candidates and molecular structures and mechanisms of action. The uncovering of novel susceptible pathways and targets interacting to the drugs, drug repositioning will be facilitated. AI-driven drug discovery leverages advanced algorithms and machine learning techniques to enhance the pharmaceutical development process. By analyzing vast datasets, including genomic information, chemical properties, and clinical outcomes, AI can identify potential drug candidates more efficiently than traditional methods [184]. Computational approaches, such as molecular modeling and simulations, enable researchers to predict interactions between drugs and biological targets, optimizing lead compounds before synthesis. These technologies streamline the identification of novel therapeutics, reduce time and costs, and improve the success rate of drug development. Thereby, AI is transforming the landscape of medicine, accelerating the delivery of innovative treatments to patients. Collaborative research includes common research between government, industry and academia by data, resources, and expertise sharing. Collaborative research in global virus control involves partnerships among scientists, governments, and organizations to share data, resources, and expertise. This interdisciplinary approach increases the understanding of viral transmission, vaccine development, and outbreak response, aiming to improve public health and decrease the burden of infectious diseases worldwide [185]. In addition, novel techniques and approaches have emerged in recent years, such as network pharmacology, artificial intelligence and machine learning, structural bioinformatics and phenotypic screening [186–188]. The complex interactions between diseases, targets and drugs are evaluated by network pharmacology to identify potential drug candidates. The analysis of large-scale biological and chemical data using algorithms of machine learning results in predicting drug-disease associations, prioritizing potential candidates and discovering novel drug targets. Molecular docking and virtual screening using structural bioinformatics techniques also predict the binding affinity of drug to the target and molecular interactions [186–188]. By combining advanced computational methods, experimental approaches, and multidisciplinary expertise, researchers can uncover promising drug candidates for repositioning with the potential to improve patient outcomes and public health.

Furthermore, the development of effective vaccines, diagnostic tools, and antiviral therapies is crucial for controlling these infectious agents. Achieving control and prevention goals greatly benefits from international collaboration, which involves sharing data, expertise, and resources among countries. It is essential to educate the public and raise awareness about the risks posed by viral agents. By promoting public awareness campaigns, individuals can be encouraged to take preventive measures, such as practicing good hand hygiene, wearing masks, and avoiding contact with wild animals. In 2018, the World Health Organization (WHO) unveiled the concept of “Disease X” as a key component of its preparedness planning for unforeseen events. This hypothetical pathogen, referred to as a ‘knowable unknown,’ serves as a potential trigger for future epidemics or pandemics. The inclusion of “Disease X” in the WHO’s list of emerging infectious diseases underscores the necessity for proactive measures that extend beyond known pathogens. The WHO is actively engaged in research and preparedness for “Disease X” with the objective of developing effective interventions and response strategies that can be quickly deployed in the event of an outbreak of a new or unknown disease [189, 190]. Another noteworthy area is artificial intelligence, which has the potential to expedite drug discovery and repositioning. By integrating in silico protocols into the initial phases of drug research and development, it becomes feasible to cut down on costs and accelerate the drug discovery process. In silico methods play a crucial role in identifying potential drug candidates with predicted therapeutic effects while screening out substances with anticipated toxicity or unfavorable pharmacokinetics. With the various hurdles in the way of current antiviral drug discovery, such as the need to develop new drugs during outbreaks and address drug resistance resulting from rapidly accumulating viral mutations, the emergence of artificial intelligence and machine learning (AI/ML) methods could hasten the pace of anti-infective drug discovery and potentially decrease overall development costs. Several drug databases, with the potential for drug repurposing, have been developed in conjunction with target resources [191, 192, 193].

## Data Availability

Data sharing is not applicable to this article as no data sets were generated or analyzed during the current study.
